# Behavior change wheel as a tool to promote physical activity in online intervention: a case study

**DOI:** 10.3389/fpsyg.2025.1498351

**Published:** 2025-04-02

**Authors:** Nuno Couto, Vitor Morgado, Tomás Pereira, Anabela Vitorino, Teresa Bento, Susana Alves, Pedro Duarte-Mendes, Luís Cid

**Affiliations:** ^1^Sport Sciences School of Rio Maior (ESDRM), Santarém Polytechnic University, Rio Maior, Portugal; ^2^Research Center in Sports Sciences, Health Sciences and Human Development (CIDESD), Vila Real, Portugal; ^3^Sport Physical Activity and Health Research and Innovation Center (SPRINT), Santarém, Portugal; ^4^Department of Sports and Well-being, Polytechnic Institute of Castelo Branco (IPCB), Castelo Branco, Portugal

**Keywords:** physical activity, behavior change, intervention, behavior change wheel (BCW), behavior change techniques (BCTs)

## Abstract

**Introduction:**

Physical activity (PA) has significant health benefits. However, one in four adults does not meet the globally recommended levels of PA. Considering that PA behavior is influenced by various factors operating at multiple levels, including personal, social, and environmental factors, a single-subject behavioral change intervention was developed to promote PA behavior through online sessions.

**Method:**

Based on a one-to-one intervention, the Behavior Change Wheel (BCW) methodology was used to design an intervention that was developed for 24 weeks, including eight weeks of online sessions and 16 weeks of follow-up.

**Results:**

We observed an increase of metabolic equivalent (MET-min/week; baseline = 2,970; eight weeks = 6,440; 24 weeks = 6,744) and daily steps (baseline = 8,372; eight weeks = 10,234; 24 weeks = 12,142), which provides some evidence for the efficacy of online methodologies, especially in the relation one-to-one.

**Conclusion:**

The intervention, designed through BCW, had a positive effect in promoting more PA in a subject of our case study and provided evidence that can be used in further interventions that aim to increase PA levels in the population; however, future studies must be conducted to expand the evidence in other domains.

## 1 Introduction

Physical activity (PA), which is defined as any bodily movement produced by skeletal muscles that requires energy expenditure, and refers to all movement including during leisure time, for transport to get to and from places, or as part of a person’s work or domestic activities (WHO, 2024), has significant health benefits for the heart, body, and mind; contributes to preventing and managing noncommunicable diseases, such as cardiovascular diseases and cancer; and reduces symptoms of depression and anxiety (WHO, 2024). However, globally, one in four adults does not meet the globally recommended levels of PA, and insufficient PA is considered the fourth leading risk factor for global mortality (WHO, 2018, 2024). The global cost of physical inactivity in direct healthcare is estimated at 54 billion dollars per year, with an additional 14 billion dollars attributable to lost productivity (WHO, 2018). Across its many forms, PA has multiple health, social, and economic benefits (WHO, 2018). Despite these benefits, adults have difficulty in initiating and maintaining PA behaviors. Several theories and models across disciplines have been suggested to enhance compliance with exercise routines and mitigate the aforementioned challenges (Ambros-Antemate et al., 2023). PA behavior is influenced by various factors operating at multiple levels, including personal, social, and environmental factors (Fletcher et al., 2018). Behavioral and social approaches aim to teach people the behavioral management skills necessary for the successful adoption and maintenance of behavior change and to create organizational and social environments that enable and enhance behavioral change (Heath et al., 2012).

The Behavior Change Wheel (BCW; Michie et al., 2014) is a comprehensive framework for designing and implementing behavior change interventions. It is a synthesis of 19 behavior change frameworks that draw on a wide range of disciplines and approaches and provides a systematic and transparent methodology for promoting behavior change (Michie et al., 2011, 2014). This method differs from others since it integrates individual, environmental, and policy-level factors, emphasizes a broad range of influences on behavior, and provides practical guidance for designing interventions across various contexts (Michie et al., 2011). This methodology is used to develop interventions that target change at three levels: policies, interventions, and behavior change techniques (BCTs; Tombor and Michie, 2017) and defines behavior as an interaction between capability, opportunity, and motivation (COM-B model: Michie et al., 2014).

Within this framework, methods are conceptualized at three levels: policies that represent high-level societal and organizational decisions; interventions that are more direct methods to change behavior, and BCTs, which are the smallest components on their own and have the potential to change behavior (Tombor and Michie, 2017). The center of the wheel is represented by COM-B, which is in line with Murphy et al. (2023) posit that changing any behavior of an individual, group, or population involves changing one or more of the following: Capability, Opportunity and Motivation. Capability can be physical or psychological; opportunity refers to factors in the physical or social environment that facilitate or hinder behavior change; and motivation can be reflective (involving conscious evaluations and planning) and/or automatic (unconscious emotional responses, impulses, or habits; Murphy et al., 2023).

Within COM-B’s components that generate behavior, it is possible to further distinguish capability, opportunity, and motivation using the Theoretical Domains Framework (TDF); the next layer of the BCW method, which allows an exploration of the barriers and facilitators of change. TDF is an integrative framework synthesizing key theoretical constructs used in relevant theories in behavior change domain. The framework consist of 14 domains: knowledge, skills, memory, attention, decision-making process, and behavioral regulation are associated with COM-B Capability; TDF environmental context and resources and social influences are associated with COM-B Opportunity; and TDF social/professional role and identity, beliefs about capabilities and consequences, optimism, intentions, goals, reinforcement, and emotion are associated with COM-B Motivation (Michie et al., 2014).

Training, enablement, modeling, environmental restructuring, restrictions, education, persuasion, incentivization, and coercion are the nine intervention functions that constitute the layer surrounding the COM-B model and TDF. Intervention functions are broad categories of things one can do to change behavior and are designed to change the capability, opportunity, and/or motivation to engage in the behavior (West and Michie, 2019). The last layer consists of seven Policy Categories: fiscal measures, guidelines, environmental/social planning, communication/marketing, legislation, service provision, and regulations.

This method guided us through a systematic, step-by-step process, moving from identifying and selecting the relevant target behaviors to a thorough analysis of these behaviors and the barriers to change according to the COM-B model, to identify the most promising, feasible, and culturally appropriate BCTs, intervention functions, and modes of delivery (Murphy et al., 2023).

Identifying intervention functions and policy categories, BCTs, and modes of delivery requires judgment as to what is most appropriate for the context, since the intervention may not be possible to implement because of context and limitations such as the mode of delivery of the intervention and resources (Michie et al., 2011, 2014; Tombor and Michie, 2017).

In summary, the BCW offers a structure plan for intervention design, the COM-B model helps to identify the barriers to behavior, and BCTs provide actionable steps to implement the intervention. Moreover, the TDF enhances this process by identifying deeper psychological and contextual factors that each part of the COM-B.

BCW has been used to promote PA behavior in several studies. Ojo et al. (2019) found that BCW can be successfully applied through a systematic process to understand the drivers of office worker behavior and develop a co-created intervention to promote breakup and decrease sitting in the workplace. Moreover, Haley et al. (2023) identified that BCW has potential mechanisms of action that improve PA in adults with spinal cord injuries. The BCW also enabled the systematic and comprehensive development of multicomponent interventions to promote PA following a community pulmonary rehabilitation program in patients with chronic obstructive pulmonary disease (Haley et al., 2023). However, no study has been conducted on healthy adults who change their daily routines to test the possible efficacy of this method in this domain.

Therefore, considering the physical inactivity of the among adults, the urgent need to identify effective methodologies for promote PA behaviors, and the potential of the BCW to design online interventions, this study aimed to develop and implement a BCW-based intervention to enhance and improve PA behavior in a single subject. To achieve this, a case study, which is a systematic investigation of an individual, group, community which the researcher examines in-depth data relating to several variables (Woods and Catanzaro, 1988) was employed.

## 2 Methods

### 2.1 Participant

This study used a single-participant A-B design (pre-test/post-test). Stein et al. (2012) state that this methodology is employed when working with a sample size of one or when a group comprises a few individuals, to establish an experimental condition in which the participant(s) serve as their own control.

The following inclusion criteria were established: adult subjects with an age range between 18 and 65 years old; physically inactive according to WHO’s guidelines; without severe medical or psychological conditions; with access to the internet and a device to participate in online sessions and availability to participate in the sessions within the period established for the program.

This intervention was performed with a 28-year-old female mother of a three-years-old child, with normal body mass index (i.e., 24.17 k/m^2^) and previous dropout episodes of structured PA participation in fitness context, obtained by convenience. Maria was invited to participate in this study due to her accessibility, willingness to dedicate herself to this intervention, and fulfillment of all the inclusion criteria. To protect the identity, the participant was identified with the nickname of Maria. Written informed consent was obtained from the participant, and this study followed ethical standards in line with the 2013 update of the Declaration of Helsinki (WMA, 2013).

### 2.2 Instruments

Yamax Digi-walker DW-500 pedometer (Yamax Corp., Tokyo, Japan), which has demonstrated scientific evidence of accuracy in measuring the number of steps and other associated measurements (Bassett et al., 1996; Bassett, 2000; Lee et al., 2015; Tudor-Locke et al., 2002). The daily average steps were calculated with registration of daily steps during 7 days.

The Portuguese version of the International Physical Activity Questionnaire (IPAQ), validated in 12 countries (Craig et al., 2003), was used to enable the quantification of PA in daily life. This questionnaire allows for the evaluation of weekly PA in walking, moderate activity, and vigorous activity. The IPAQ categorizes PA according to the frequency and duration of each specific type of activity and the time spent seated each day of the week. We converted the data obtained from the IPAQ to metabolic equivalent (MET)-min/week by calculating the marked minutes per week in each category of activities by the specific metabolic equivalent. We then classified the participant PA levels based on the IPAQ recommendations into the following categories:

Category 1 (Low): The lowest PA level corresponds to individuals who do not fulfill the criteria for Categories 2 or 3. The IPAQ considers these individuals inactive.

Category 2 (Moderate): Corresponds to individuals who meet one of the following criteria: (a) participated in three or more days of vigorous physical activity for at least 20 min a day; (b) participated in five or more days of moderate PA and/or walking for at least 30 min a day; (c) participated in five or more days of any combination of walking, moderate, or vigorous physical activity, and reached a minimum total physical activity of at least 600 MET-min/week.

Category 3 (High): Consists of individuals who meet one of the following criteria: (a) participated in vigorous activity for at least three days per week, reaching a minimum total PA of 1,500 MET-min/week; (b) participated seven days a week in any combination of walking and moderate or vigorous activities, and reached a minimum total PA of at least 3,000 MET-min/week.

### 2.3 Procedures

Before intervention, an initial assessment of PA behavior, where the IPAQ and pedometer were used, and a semi-structured, one-on-one interview were conducted in October 2023. This moment allowed us to tailor the behavior change intervention. The interview aimed to explore Maria’s routine, providing essential information to design an intervention based on the BCW framework. A preliminary interview was created based on a review of this model. A panel of experts (i.e., psychologists, sport and health researchers) reviewed the guide to assess the question clarity, alignment with the study goals, and comprehensiveness.

Maria was interviewed in a quiet environment, and the interview was recorded with her consent. The semi-structured format allowed for flexibility, enabling follow-up questions based on her responses. Throughout this moment, the participant’s routine was explored to provide information about her routine and challenges, which was essential for designing the online intervention. After the initial moment, the intervention was designed and implemented using step-by-step guidance from the BCW, as suggested by Michie et al. (2014), and was categorized into three stages over eight steps. The pedometer was the only instrument that continued to be used by the subject in an autonomous way in subsequent assessments how long the program. The intervention was created over the course of 24 weeks, as identified in Table 1. There was an eight-week period in which Maria followed a structured PA routine that involved two weekly workouts of resistance exercise, and three 30-min walks. Throughout these weeks, a trained exercise technician provided online support (via the Zoom platform) for the planned routine. The technician did not directly participate in the three weekly walks.

**Table 1:** Study intervention phases and timeline.

Initial assessment	Online sessions	Follow-up	Final assessment
October 2023	8 weeks	16 weeks	March 2024
Semi-structured interview. Total Metabolic Equivalent (MET) in a week of PA (IPAQ). Mean of Daily Steps in a Week (Yamax Digi-walker DW-500 pedometer).	Prescribed training monitorization. BCTs Implementation.	Behavior monitorization (i.e., exercise twice a week at home and three times 30 min)	Total Metabolic Equivalent (MET) in a week of PA (IPAQ). Mean of Daily Steps in a Week (Yamax Digi-walker DW-500 pedometer).
	24 weeks		

Following the first eight weeks, Maria completed the prescribed exercise and walks on her own for a total of 16 weeks. This time-bound approach permits six months of intervention, which is the temporal window within which individuals can reach the maintenance stage in the Transtheoretical Model (Prochaska and DiClemente, 1982, 1983). The 60-min resistance exercise workout that Maria followed in this intervention was created in accordance with the exercise adults recommended for ACSM (2021). This workout consists of three sets of 12 repetitions split between upper and lower body exercises, and it includes warm-up and cool-down phases.

Moreover, Maria was educated about the instructions of the Borg’s CR-10 scale (Borg, 1998) for intensity control in resistance exercise workouts and walks. The rating of perceived exertion (RPE) scale is a perceptual-based assessment method that uses a combination of numbers and verbal descriptors. On the scale, category 10 corresponds to maximum exhaustion and zero to rest. Maria was asked to maintain the intensity in these moments between three and seven, the comprehensive interval intensity between moderate and vigorous intensity, according to Zuhl (2020).

To facilitate efficient contact between Maria and the technician during this time, a communication toll was also made available via the WhatsApp platform.

## 3 Results

### 3.1 Stage 1: Understand the problem and the behaviors

#### 3.1.1 Step 1: Define the problem in behavioral terms

Through the initial phase of the intervention, Maria took an average of 8,370 steps per day. In line with the study by Paluch et al. (2022), considering the participant’s age, this average number of steps is adequate to reduce all causes of mortality; however, it is below the number of steps suggested as low risk (i.e., 9,000–10,500; Ahmadi et al., 2024). It was also observed that this number is essentially justified for work tasks that are categorized as low intensity, and no activities with moderate to high intensities were identified. Thus, considering the PA guidelines of the WHO (2024) and ACSM (2021) for adults, which recommend at least 150 to 300 min of moderate aerobic activity per week or the equivalent vigorous activity and muscle-strengthening activities at moderate or greater intensity that involve all major muscle groups on two or more days a week, we can conclude that the participant failed at this point. Despite the daily amount of PA, Maria aims to adhere to and maintain participation in structured PA activities, as various dropout episodes of sportive practice were identified during her life in a fitness context as, she identified: “I have already tried sometimes to do exercise in the gym but I cannot maintain this activity.” Maria also identified that this situation was derived from disinterest in activities, the context of practice, and a lack of time. The adherence and maintenance of the structured PA practice could increase the intensity of PA to achieve the global recommendations by WHO (2024).

#### 3.1.2 Step 2: Select target behavior

To reduce all causes of mortality and promote mental and physical health, the recommendations of PA for healthy adults are convergent into type, time, and frequency (WHO, 2024); and, considering the previous point, the need to promote moments in the Maria’s life of structured PA that include aerobic, and resistance exercises as recommended by ACSM and WHO seems clear. To select the target behavior among the potential aerobic and resistance exercises, it is essential to choose the behavior that meets the four rating criteria suggested by Michie et al. (2014): (I) how much of an impact changing the behavior will have on the desired outcome; (II) how likely is it that the behavior can be changed (when considering the likelihood of change being achieved, think about the capability, opportunity, and motivation to change of those performing the behavior); (III) how likely it is that the behavior (or group of behaviors) will have a positive or negative impact on other, related behaviors; and (IV) how easy will it be to measure the behavior. Based on the recommendations of PA for healthy adults and considering the actual behavior of the participant, the practice of structured PA was targeted at a minimum of 30 min of moderate-to-intensive intensity at least 5 days a week to increase the general PA in her routine. Therefore, it was prescribed a resistance exercise workout twice a week, with 60 min of duration each session, and three 30-min walks.

#### 3.1.3 Step 3: Specify target behavior

As suggested by Michie et al. (2014), after selection, the necessary behavior is specified. Therefore, we answered the following questions to specify the target behavior as demonstrated in Table 2.

**Table 2:** Specifying the target behavior.

Target behavior	The practice of structured PA a minimum of 30 min of moderate-to-intensive intensity at least 5 days a week.
Who needs to perform the behavior?	Considering the previous points, Maria needs to increase the amount and intensity levels of the PA.
What does she need to do differently to achieve the desired change?	Based on COM-B diagnosis, it is important to increase PA in Maria’s life, through leisure moments in her daily routine.
When does she need to do it?	During the week, Maria will perform some alterations on her daily routine to increase PA amount and implements a routine of resistance exercise workout twice weekly and three 30-min walk, three times per week, with moderate to intensive intensity.
Where does she need to do it?	Maria will make alterations to PA behavior at work and at home.
How often does she need to do it?	Maria will perform this behavior daily.
With whom does she need to do it?	Coworkers, family, and friends.

#### 3.1.4 Step 4: Identify what needs to change

Based on the behavioral diagnosis, we identified the barriers and facilitators to Maria becoming more physically active in terms of capability, opportunities, and motivation. Table 3 shows the changes in behavioral terms to help Maria achieve the target behavior.

**Table 3:** Behavioral analysis and diagnosis.

Target behavior	The practice of structured PA a minimum of 30 min of moderate to intensive intensity at least 5 days a week.	
COM-B components	What needs to happen for the target behavior to occur?	What is necessary to the behavior change?
Physical capability	Maria needs to have physical conditions to be more PA active.	Maria considers that having enough physical capacity improves PA behavior. Through PARQ+, it was also identified that Maria has no limitations or health restrictions for PA practice.
Psychological capability	Maria does not know the correct techniques and skills to perform exercises autonomously.	Maria needs to learn more techniques to perform the PA routines autonomously, since she identified “I do not know how to do the resistance exercises alone. When I was in the gym, I always needed help from the technician.” Change is needed.
Physical opportunity	Maria needs to have the opportunity to be active in different physical contexts in Maria’s life.	Maria identified that she has the conditions, space, and materials to do PA at home in her neighborhood and at her workplace, since she identified that “I have great conditions near home and workplace for walk safety. And, at home I have fitness equipment (e.g., dumbbells) and good conditions for exercise.” However, she needs help planning her PA.
Social opportunity	It is important to see members of close social networks valuing physical activity and getting active.	Maria identified that “I have friends, family, and coworkers that also would like to improve their PA.” However, she needs help to implement strategies to involve those in this activity. Change is needed.
Reflective motivation	Maria needs to have and hold beliefs that being physically active impacts various facets of health.	Rising beliefs that Maria has enough capacities to join a specific program of PA and that it is beneficious for her. Change is needed.
Automatic motivation	Maria needs to establish routines and habits for the practice of physical activity.	Change is needed to establish habits and routines for PA practice since Maria does not have this habit. “I would like to workout regularly. I cannot maintain the exercise routine during time.”
Behavioral diagnosis	Psychological capability, social opportunity, physical opportunity, reflective, and automatic motivation needed to be changed to improve the PA behavior of Maria.	

### 3.2 Stage 2: Identify intervention options

#### 3.2.1 Step 5: Identify potential interventions functions

All nine intervention functions were selected for the intervention: education: increasing knowledge and understanding by informing, explaining, showing, and correcting; persuasion: changing the way people feel about a behavior by generating cognitive dissonance and showing how changing behavior can reduce it; incentivization: changing the attractiveness of a behavior by creating the expectation of a desired outcome or avoidance of an undesirable one; coercion: changing the attractiveness of a behavior by creating the expectation of an undesired outcome or denial of a desired one; training: increasing psychological or physical skills, or habit strength by explanation, demonstration, practice, feedback, and correction; environmental restructuring: constraining or promoting behavior by shaping the physical or social environment; modeling: showing examples of the behavior for people to imitate; and Enablement: providing support to improve ability to change in a variety of ways not covered by other intervention functions (West and Michie, 2019).

#### 3.2.2 Step 6: Identify policy options

Because this intervention was performed using a single-participant A-B methodology, policy options were not applied.

### 3.3 Stage 3: Identify content and implementation options

#### 3.3.1 Step 7: Identify BCT’s

After the initial diagnosis using the COM-B model, the intervention was developed and applied to the BCT’s as shown in Table 4.

**Table 4:** COM-B model, TDF domain, intervention functions, policies; BCTs.

COM-B component and TDF	Formative assessment	Intervention functions	BCTs	BCTs implementation
Psychological capability (Knowledge; cognitive and interpersonal skills; memory; attention and decision process; behavioral regulation)	Maria knows what PA is and its benefits. Maria does not know the correct techniques and skills to perform exercises autonomously.	Training Enablement.	Demonstration of the behavior. Instruction on how to perform a behavior. Behavioral practice/rehearsal. Feedback on the behavior. Goal setting. Problem solving. Action planning. Self-monitoring of behavior. Restructuring the physical environment.	It was identified, regarding this dimension, the need to enhance the skills of Maria for exercise training to be able to follow, in autonomy, the prescribed training plan. Thus, the identified BCTs were implemented through a practical explanation of the exercises and critical components of the same. Maria performed/rehearsed the prescribed exercise; there was feedback to optimize the knowledge, cognitive capacity, and regulation of behavior in relation to the prescribed training.
				It was defined that Maria would perform twice weekly the prescribed training, with online supervision during eight weeks. All sessions were also recorded, which allowed Maria to check her performance and send us her feedback about exercises. The training was also prescribed considering the fitness material that Maria has at home and the need for adequate space in her living room for her exercise safety.
Physical capability (Physical Skills)	Maria realizes that she has the physical capacity to increase the intensity and volume of PA.	*	*	*
Social Opportunity (Social Influences)	Maria identified that the practice of PA with others is more enjoyable. Maria has good support from family and friends for PA practice, and she has coworkers interested in adhering to PA.	Enablement.	Social support Goal setting (behavior) Adding objects to the environment Problem solving Action planning Restructure the physical environment	Considering Maria’s social network, she proposed a walk with some of her coworkers, three times per week, during 30 min of lunchtime in the workplace area. A specific road map was created to allow Maria and her colleagues to walk safely during their free lunch break. Furthermore, a WhatsApp group was formed to promote an effective tool for communication among them. At home, Maria’s husband was an ally to guarantee support and helped Maria to achieve the previously defined goal.
Physical Opportunity (Environmental Context and Resources)	Maria has fitness facilities near her home to join. However, considering her past in this context, she is more proficient in promoting other contexts to PA practice. Maria’s husband has some fitness material that she can use at home. There is a nearby workplace with outdoor conditions to perform PA.	Environmental Restructuring Enablement.	Restructuring the physical environment Problem solving Action planning	Maria has places, for example, forest trails near home and her workplace that allow her to go for walks. The trail road map was adapted to be in harmony with the time available and the physical conditions of Maria. At home, the fitness equipment of her husband was collected in a specific room for Maria to do the resistance workouts. Adaptation of home training plans considering the available fitness equipment and surrounding spaces.
Reflective Motivation (Professional/Social role and identity; Beliefs about capabilities; Optimism; Beliefs about consequences; Intentions; Goals)	Maria does not have the confidence to maintain the PA routine. Believe that her sedentary behavior is related to a lack of time. Negative beliefs based on previous attempts at PA adherence. Underestimate the present behavior and its consequences for health.	Education Persuasion Incentivization Coercion Modeling.	Feedback on behavior. Information about health consequences Feedback on the outcome of the behavior. Self-monitoring of behavior.	Through feedback on behavior, we noticed she has not achieved the PA recommendations. We also identified the consequences of it for her health. Maria was also informed that her physical condition and body composition will get better whether PA behavior is improved. We had to show Maria inspirational stories of others who changed their lifestyles and became more physically active, improving the beliefs. Between session one and session three, Maria was incentivized to do, for 1 week, three little walks and register them.
Automatic Motivation (Reinforcement; Emotions)	Currently, Maria does not have the habit of regular practice of the PA.	Training; Environmental Restructuring Enablement.	Prompts/cues Instructions on how to perform the behavior Self-monitoring of behavior. Social support Goal setting (behavior) Adding objects to the environment Problem solving	To promote the walk habit, and after the formation of a colleague group, everybody was notified by smartphone on walk days. Maria had a calendar where she recorded all walks. The communication between all colleagues to walk was made through a WhatsApp group. On the days that Maria exercises at home, her husband sends her a text message and develops the household tasks that Maria usually does.

*Not applicable.

#### 3.3.2 Step 8: Mode of delivery

According to Michie et al. (2014), the mode of delivery can be face-to-face or distant. Considering the practicality and the possibility of quick access to online tools, the intervention was designed and implemented using an online methodology.

## 4 Discussion

Through an initial diagnosis powered by the COM-B model, a behavior change intervention was designed and implemented, which resulted in PA improvement in Maria. She underwent BCTs during her routine eight weeks of intervention and 16 weeks of follow-up period. In total, we checked Maria’s PA behavior for 24 weeks, a time-bound period that encompassed 6 months. According to Prochaska and DiClemente (1982, 1983), this is considered the temporal window to achieve the maintenance stage of the Transtheoretical Model, and research has also shown that the application of some BCTs (e.g., action planning and self-monitoring) is essential for increasing and maintaining PA behavior for more than six months (Murray et al., 2017). This intervention consisted of the application of BCTs that were applied according to the COM-B diagnosis, and it allowed Maria to increase daily steps and metabolic expenditure of PA, as indicated in Table 5. Moreover, Maria succeeded in the maintenance of resistance exercise workouts twice a week and three 30-min walks a week during the intervention, including the follow-up period. It is assured that Maria achieves the main recommendations of PA for adults by ACSM (2021), since she completes at least 150 to 300 min of moderate aerobic activity per week or the equivalent vigorous activity and muscle-strengthening activities at moderate to vigorous intensity that involve all major muscle groups on two or more days a week. This increment of PA aligns with the study goal and can potentiate health and disease prevention, since those who achieve total PA levels several times higher than the current recommended minimum level have a significant reduction in the risk of some diseases, such as breast cancer, colon cancer, diabetes, ischemic heart disease, and ischemic stroke events (Kyu et al., 2016). The COM-B model is a theoretical framework that identifies the key factors influencing behavioral change, including capability, opportunity, and motivation (Michie et al., 2011). These components are interlinked, and increasing opportunities or capabilities can increase motivation. Expanded motivation can lead people to do things that will increase their capability or opportunity by changing their behavior.

**Table 5:** PA outcomes assessment.

Baseline	8 weeks	24 weeks
Total metabolic equivalent (MET) in a week*		
2,970	6,440	6,744
Mean of daily steps in a week**		
8,372 ± 1345.60	10,234 ± 1460.70	12,142 ± 2130.62

*Assess by IPAQ; **assess by pedometer.

The capability component refers to an individual’s psychological and physical ability to participate in a specific activity and encompasses the skills, knowledge, and abilities required to perform a behavior (Michie et al., 2011; Willmott et al., 2021). Therefore, through the COM-B diagnosis, it was identified that change is needed in psychological capability, not in the physical capability domain. According to Michie et al. (2011), physical capability can be achieved through physical skill development, which is the focus of training, or by enabling interventions such as medication, surgery, or prostheses. Thus, considering that physical limitations for PA practice were not identified in the initial assessment, it was defined that this component would be out of the behavior change process.

However, their psychological capabilities must also be improved. This component refers to the capacity to engage in the thought processes, comprehension, and reasoning necessary to perform the target behavior (Michie et al., 2011). It is characterized by knowledge or physical skills, strength, or stamina to engage in the necessary mental process and can be achieved by imparting knowledge or understanding, training emotional, cognitive, and/or behavioral skills, and is associated with the following TDF: knowledge, memory, attention and decision processes, and behavioral regulation (Michie et al., 2011, 2014). Maria was aware of the importance of PA for her health and was familiarized with this intervention. Moreover, education on the benefits of PA is not essential for promoting PA behavior. Barrett et al. (2022) identified in a study of adults that increased knowledge was not described as a key component needed by participants to increase PA. In this study, the participants repeatedly stated that they did not need to be told that they would benefit from increasing PA since someone who is unfit knows that they need to get fit. However, related to this component, Maria understood that she needed to increase her PA behavior. We also identified that Maria needed to improve her skills to properly perform the resistance exercise workout program designed for her, as she had identified that she could not perform the prescribed exercises without supervision, especially because of her lack of knowledge about the execution of some exercises. Hence, through the intervention functions of training and enablement, various BCTs were implemented (Table 4) to promote Maria’s knowledge, behavioral regulation, and skills related to the prescribed exercises. Psychological capability has been linked to motivation and is predictive of moderate-to-vigorous PA behavior in adults (Howlett et al., 2019). According to Willmott et al. (2021), psychological capability is associated with physical activity behavior, both directly and indirectly, through its impact on motivation. These authors found that in the context of young adults’ physical activity and eating behaviors, young adults who believed they were capable, had control, held favorable attitudes, and/or intentions of being physically active or eating healthy, and were more motivated to engage in the behavior identified as essential to endorsing psychological capability in the promotion of healthy behaviors.

It was also identified that both social and physical opportunities should be endorsed to promote behavioral change in Maria’s PA. The opportunity component can be “physical” (what the environment allows or facilitates in terms of time, triggers, resources, locations, physical barriers, etc.) or “social” (including interpersonal influences, social cues, and cultural norms; Chen et al., 2022), and it could be achieved through environmental change (Michie et al., 2011).

Therefore, regarding this component and in line with the COM-B diagnosis that allows identification of the barriers and facilitators of behavior, it was suggested that some changes in Maria’s social and physical context contributed to PA promotion, as identified in Table 4. Regarding social opportunities, several BCTs were implemented to increase social support for PA practice in the workplace and at home, as general social support and support from family members were positively associated with leisure-time PA (Garcia et al., 2022). In general, promoting social opportunities, such as social support, norms, and group membership, has been positively related to PA behavior (Chen et al., 2024; Garcia et al., 2022; Seims et al., 2023; Stevens et al., 2017). Willmott et al. (2021) wrote that opportunity as a component holds environmental cues and resources that either encourage or inhibit behavior and might involve factors such as time availability, access to resources, and social support. According to Chen et al. (2024), it is important to identify the factors of work and/or home environments that support or hinder the maintenance of regular physical activity. Therefore, to address physical opportunities, and considering that Maria identified that in her environment, we realized that she has conditions to implement PA but needs help to plan it. As indicated in Table 4, the following BCTs: restructuring the physical environment, problem solving, and action planning, allowed us to expand the available resources to improve Maria’s PA, diminish the barriers, and improve the facilitators of PA behavior.

Finally, the study identified that reflective and automatic motivations need to be adjusted. Agreeing to Michie et al. (2014), reflective motivation is a process involving plans (self-conscious intentions) and evaluations (beliefs about what is good or bad). Despite Maria’s self-awareness of her PA behavior and understanding that PA is beneficial for her health, she did not achieve the recommended amount of PA. Hence, we implemented the following BCTs (Table 4): feedback on behavior, information about health consequences, feedback on outcome behavior, and self-monitoring of behavior, techniques that reinforced Maria’s beliefs and intentions regarding PA. Beyond the BCTs that allowed the assessment of actual behavior and improved self-awareness of it (i.e., feedback on behavior, information about health consequences, and feedback on outcome behavior), the following BCT self-monitoring of behavior was implemented to facilitate the incentivization of PA. This technique encompasses keeping a record of behavior, more specifically Maria’s PA activity behavior. It helps individuals become more aware of their physical activity levels and encourages them to set goals, track progress, and receive feedback. This technique has been positively associated with improving PA in adults (Compernolle et al., 2019; Gardner et al., 2016), and has been linked to increasing PA from moderate to vigorous (Larsen et al., 2022). The benefits of this technique are better when it involves complex interventions combining self-monitoring with other components (Vetrovsky et al., 2022). Thus, regarding reflective motivation, we applied various techniques that improved Maria’s intentions and beliefs about PA and asked Maria to register her PA behavior frequency during this intervention (i.e., resistance exercise workouts and three 30-min walks).

It was also found that this intervention with Maria required a change in automatic motivation since she did not have PA habits. This component is characterized by an automatic process involving emotions and impulses that arise from associative learning and/or innate dispositions (Michie et al., 2011). The development of habit formation is long and requires many repetitions of the same behaviors in the same context (Lally and Gardner, 2013) and the success of habit formation depends on the frequency and contextual cues associated with a response (Wood and Neal, 2009). Therefore, some of the techniques identified in Table 4 were implemented to promote automatic behaviors. Smartphones/notifications were used to develop prompts/cues through calendar application notifications to determine walking days during all interventions at lunchtime at Maria’s workplace, including during the follow-up period. In fact, research has found that text messaging can help form PA habits in the workplace and might facilitate the long-term maintenance of PA behaviors (Fournier et al., 2017). In addition, Maria was asked to register the frequency of behaviors to achieve previously determined goals for PA activity behaviors (walking in the workplace and workouts at home). According to Michie et al. (2013), self-monitoring of behavior is an established method for a person to monitor and record their behavior (s) as part of a behavior change strategy. This technique can lead to self-awareness regarding behaviors and help individuals regulate their behavior more effectively by avoiding and coping with situations that often lead to failure (Burgard and Gallagher, 2006). Social support and problem-solving were also implemented to promote automatic motivation. Problem solving, analyzing, or prompting the person to analyze factors influencing behavior and generating or selecting strategies that include overcoming barriers and/or increasing facilitators (Michie et al., 2013), and social support, advising on, arranging, or providing social support (e.g., from friends, relatives, colleagues,’ buddies,’ or staff), have been seen as promoting PA behavior (Gilchrist et al., 2024). Problem-solving strategies can enhance self-efficacy for PA and have been positively associated with promoting PA behaviors (Olander et al., 2013). Higher levels of social support can lead to increased opportunities for social interactions, positively influence health behavior decision-making, enhance access to resources related to PA (Cao et al., 2023), and potentiate predictors, such as motivation and attitude, that aid the process of making PA automatic driving by repeating the behavior at a more automatic level (Hopkins et al., 2022).

To the best of our knowledge, this study is the first to perform both the diagnosis and implementation of an intervention to promote PA behavior based on the BCW method of Michie et al. (2014). Despite the positive results of this intervention, we consider the present case study exploratory and insufficient to determine the power of this tool to promote behavioral change in the PA domain. To overcome this limitation, we suggest, in future research, replication on a larger sample to test the utility and power of the BCW in designing tailored efficacious interventions to promote PA behavior in several domains (e.g., fitness centers or health clubs). Beyond it, the presence of the control group will strengthen the results. Moreover, it is important to apply this methodology to people with different characteristics (e.g., male, female, young, and older adults) to identify the power of different behavior change techniques in promoting PA behavior.

## 5 Conclusion

This study aimed to identify and diagnose the problematic aspects of Maria’s PA behavior and design and implement an intervention through the BCW methodology to improve her PA. After eight weeks of online sessions, Maria had already improved her PA behavior by integrating PA into her life routine (i.e., walking three times per week, and a resistance exercise workout program twice a week), which resulted in an improvement in the amount and intensity of her weekly PA, which remained after the follow-up period.

With these results, we demonstrate how the BCW methodology, applied in a simple context through online resources, serves as a comprehensive framework to ensure the promotion and change in the PA of Maria, demonstrating the utility of BCTs in the promotion of capability, opportunity, and motivation for this behavior.

Despite the limitations of this study, we can affirm that this BCW has specificities that can help professionals in field design and implement, with success, tailored interventions to promote PA behavior.

## Data Availability

The raw data supporting the conclusions of this article will be made available by the authors, without undue reservation.
